# A study on the prevalence and influencing factors of the willingness of healthy subjects to participate in clinical trials

**DOI:** 10.3389/fpubh.2026.1735641

**Published:** 2026-02-27

**Authors:** Zhen Shen, Tao Wang, Fang-Fang Liu, Qi-Qiong Ding, Xiao-Qin Liu, Qing Li, Yi-Jun Zhang, Lian-Lian Fan

**Affiliations:** 1 Phase 1 Clinical Trial Center, Deyang People's Hospital, Deyang, Sichuan, China; 2 Clinical Pharmacology Research Center, Chinese Academy of Medical Sciences & Peking Union Medical College, Beijing, China; 3 Health Management Center, Deyang People's Hospital, Deyang, Sichuan, China; 4 The First Affiliated Hospital of Shihezi University, Shihezi, Xinjiang, China

**Keywords:** clinical trials, epidemiology, healthy subjects, influence factor, willingness to participate

## Abstract

**Objective:**

To investigate the willingness of healthy subjects to participate in clinical trials and its influencing factors, to improve recruitment efficiency.

**Methods:**

A convenience sampling method was employed to conduct a questionnaire survey among healthy subjects using an online questionnaire system. Information on demographic characteristics, opinions on clinical trials, and willingness to participate in clinical trials was collected. Statistical description, along with univariate and multivariate unconditional logistic regression analyses, were used to evaluate the epidemiological characteristics of healthy subjects and the influence of related factors on their willingness to participate in clinical trials.

**Results:**

A total of 423 valid questionnaires were collected. The average age of the healthy subjects was 29.13 ± 8.20 years old, with a gender ratio of 0.66:1. The willingness of healthy subjects to participate in clinical trials was 53.66% (227/423). Logistic regression analysis showed that urban subjects had higher willingness than rural subjects (*OR* = 2.175, *95% CI*: 1.291–3.664). Unemployed individuals not actively seeking work subjects (*OR* = 2.891, *95% CI*: 1.134–7.371) and those with a moderate level of understanding of clinical trials (*OR* = 3.906, *95% CI*: 1.950–7.827) also showed higher willingness. In contrast, subjects who were aware of the functions of an ethics committee had lower willingness (*OR* = 0.565, *95% CI*: 0.335–0.952). Factors such as age, gender, and average monthly income were not significantly associated.

**Conclusion:**

Residence, employment, and level of understanding of clinical trials significantly influence the willingness of healthy subjects to participate. Targeted strategies such as enhancing education in rural areas, optimizing recruitment among the unemployed individuals not actively seeking work, and improving ethical communication should be implemented to increase participation.

## 1 Introduction

Clinical trials are systematic investigations conducted on human subjects (either patients or healthy subjects) to evaluate the efficacy, safety, and pharmacological properties such as absorption, distribution, metabolism, and excretion of investigational drugs or medical devices (1). Healthy subjects play an indispensable role in this process. A healthy subject refers to an individual with no known diseases or abnormalities relevant to the study, with physiological and laboratory parameters within normal ranges, free from interfering factors, who volunteers to participate and does not seek therapeutic benefit. The development and approval of any new drug depend critically on their participation. Unlike patient volunteers, healthy subjects do not suffer from the condition the drug is intended to treat. They undertake potential unknown risks without the prospect of therapeutic benefit, making their willingness to participate a central ethical and practical concern (2, 3). Therefore, understanding the factors that influence the willingness of healthy individuals to take part in clinical trials and conducting targeted educational initiatives is essential for enhancing trust and engagement.

The willingness of healthy subjects to participate directly impacts the speed and quality of trial recruitment, which in turn affects both the timeline and cost of clinical development (4). However, participant recruitment remains a major challenge in clinical research. The decision to enroll is influenced by a complex array of factors. While substantial research has focused on factors affecting patients’ willingness to participate, studies specifically addressing healthy volunteers remain relatively limited. Existing research, both internationally and domestically, has identified several influential factors, including inadequate publicity of clinical trial information (5), insufficient communication from research staff (6), as well as demographic variables such as gender and educational level (7), and trust in medical staff (8).

Despite these efforts, there remains a notable lack of empirical research within the socio-cultural context of China examining the willingness of healthy subjects to participate in clinical trials. In particular, comprehensive analyses that integrate multiple dimensions such as socio-demographic characteristics, levels of awareness of clinical trials, and understanding of ethical oversight are still scarce. To address this gap, this study employs a cross-sectional survey design within the Chinese context, specifically targeting healthy subjects – a group often underrepresented in clinical trial participation research. By recruiting individuals from a health examination center and community residents, the study aims to assess willingness to participate and identify key influencing factors unique to this population. The findings are expected to contribute to the theoretical framework of clinical trial recruitment and offer practical insights for improving recruitment efficiency, safeguarding participants’ rights, and enhancing the overall quality of clinical research.

## 2 Subjects and methods

### 2.1 Research subjects

A convenience sampling method was used. The study participants were recruited from two sources: (1) individuals attending the Health Management Center of Deyang People’s Hospital for routine physical examinations, and (2) community-dwelling residents. The recruitment period spanned from October 2024 to April 2025.

Inclusion criteria were: (a) Age and gender: healthy males and females aged 18 years or above. (b) Weight index: male weight ≥ 50.0 kg, female weight ≥ 45.0 kg, body mass index (BMI) between 19.0 kg/m^2^ and 26.0 kg/m^2^. (c) Willingness to cooperate and complete this study.

Exclusion criteria were: (a) Those with clinically significant abnormalities found during physical examination, ECG, complete blood count, blood biochemistry tests, screening for infectious diseases, vital signs, etc. (b) Those with a history or current diagnosis of major diseases affecting the circulatory, endocrine, nervous, digestive, respiratory, hematologic, immune systems, psychiatric disorders, or metabolic abnormalities. (c) Those who change their mind during the survey and are unwilling to cooperate with the study.

After signing the informed consent form, questionnaires were distributed and collected by online questionnaire system. Out of 475 questionnaires, 423 valid responses were collected, resulting in an effective response rate of 89.05%, enhancing data reliability. Based on their willingness to participate in clinical trials, the subjects were categorized into two groups: a willingness to participate group and a non-willingness to participate group.

### 2.2 Questionnaires

The questionnaire consisted of a self-made questionnaire, and the final version was formed after repeated revisions based on a pilot survey. The questionnaire was refined through repeated revisions and pilot testing, increasing the reliability and clarity of the survey instrument (Cronbach’s *α* = 0.732). Data included demographic variables, opinions on clinical trials, understanding of ethical oversight, and prior participation, providing a multidimensional perspective. The content included: (1) Demographic characteristics: gender, age, ethnicity, residence, education level, employment, marital status, average monthly income, and the number of children, etc. (2) Opinions on clinical trials: participation history of oneself/relatives and friends, rights and interests of subjects, ethics committee, etc. (3) Willingness to participate in clinical trials: “Are you considering participating in clinical trials? Yes/No”.

### 2.3 Statistical methods

After the questionnaires were double checked for logic, a database was established. Excel software was used for data organization, and SPSS 25.0 software was used for statistical analysis. If the missing data exceeds 5% of the questionnaire, it is considered as unqualified. For items with fewer missing values (<5%), mean imputation is used for continuous variables, and mode imputation is used for categorical variables. For quantitative data, normality and homogeneity of variance were assessed; accordingly, the *t* test was used for mean comparisons and the *Chi-square* test for proportions. Employing multivariate logistic regression allows identification of independent predictors of willingness to participate, controlling for confounding factor. Variables significant in univariate analysis or deemed conceptually relevant were included in the multivariate model. The significance level was set at *α* = 0.05.

## 3 Results

### 3.1 Basic information

A total of 423 valid questionnaires were recovered. The average age of the healthy subjects was 29.13 ± 8.20 years old, including 168 males and 255 females, with a gender ratio of 0.66:1. The Han ethnicity was the dominant group, accounting for 94.80% (401/423). Healthy subjects from rural areas accounted for 57.92% (245/423). Those with a college and above was the main group in terms of education, accounting for 79.67% (337/423). Unmarried and childless groups were the main groups, accounting for 60.99% (258/423) and 66.90% (283/423) respectively. The average monthly income was mainly ≥5,000 yuan (38.53%, 163/423), followed by 3,000–4,999 yuan (37.83%, 160/423), and finally <3,000 yuan (23.64%, 100/423) (see Table 1).

**Table 1:** Basic information of healthy subjects.

Variable	*N*	Mean (M)/constituent ratio (%)	Standard deviation (SD)/cumulative constituent ratio (%)
Age (years)	423	29.13	8.20
Gender			
Male	168	39.72	39.72
Female	255	60.28	100.00
Ethnicity			
Han	401	94.80	94.80
Other	22	5.20	100.00
Residence			
Urban	178	42.08	42.08
Rural	245	57.92	100.00
Education level			
Junior high school and below	34	8.04	8.04
Senior high school	52	12.29	20.33
College and above	337	79.67	100.00
Marital status			
Unmarried	258	60.99	60.99
Married	144	34.04	95.03
Divorce	21	4.97	100.00
Number of children			
Childless	283	66.90	66.90
Only one	97	22.93	89.83
≥2	43	10.17	100.00
Average monthly income (yuan)			
<3,000	100	23.64	23.64
3,000 ~ 4,999	160	37.83	61.47
≥5,000	163	38.53	100.00
Employment			
Full-time job	250	59.10	59.10
Seeking employment	56	13.24	72.34
Unemployed individuals not actively seeking work	117	27.66	100.00
Smoke			
Quit smoking	34	8.04	8.04
Smoking	38	8.98	17.02
Never smoke	351	82.98	100.00
Drink			
Quit drinking	57	13.48	13.48
Drinking	58	13.71	27.19
Never drinking	308	72.81	100.00

### 3.2 Results of the univariate analysis on predictors of willingness to participation among healthy subjects

A total of 227 healthy subjects reported their willingness to participate, resulting in a participation willingness rate of 53.66%. Univariate analysis of healthy subjects’ willingness to participate revealed statistically significant differences between the two groups with respect to the following variables: gender, residence, average monthly income, employment, level of understanding of clinical trials, history of Clinical Trials, history of clinical trials among relatives and friends, understanding the functions of an ethics committee, and when participating in clinical trials, seek advice from relatives and friends. No statistically significant differences were observed about age, ethnicity, marital status, number of children, smoke, drink, and relatives and friends work in the medical industry (see Table 2).

**Table 2:** Results of the univariate analysis on predictors of willingness to participation among healthy subjects.

Variable	*N*	Willingness to participate (*n*_1_ = 227)	Non-willingness to participate (*n*_2_ = 196)	*t/χ^2^* value	*p*-value
Age (years)	423	29.46 ± 8.24	28.73 ± 8.15	0.911	0.363
Gender				12.644	<0.001
Male	168	108	60		
Female	255	119	136		
Ethnicity				0.125	0.723
Han	401	216	185		
Other	22	11	11		
Residence				14.867	<0.001
Urban	178	76	102		
Rural	245	151	94		
Education level				21.417	<0.001
Junior high school and below	34	24	10		
Senior high school	52	41	11		
College and above	337	162	175		
Marital status				3.181	0.204
Unmarried	258	139	119		
Married	144	73	71		
Divorce	21	15	6		
Number of children				2.605	0.272
Childless	283	147	136		
Only one	97	52	45		
≥2	43	28	15		
Average monthly income (yuan)				7.509	0.023
<3,000	100	57	43		
3,000 ~ 4,999	160	96	64		
≥5,000	163	74	89		
Employment				27.892	<0.001
Full-time job	250	117	133		
Seeking employment	56	48	8		
Unemployed individuals not actively seeking work	117	62	55		
Smoke				2.850	0.240
Quit smoking	34	22	12		
Smoking	38	23	15		
Never smoking	351	182	169		
Drink				4.221	0.121
Quit drinking	57	33	24		
Drinking	58	24	34		
Never drinking	308	170	138		
Relatives and friends work in the medical industry				1.233	0.284
Yes	210	107	103		
No	213	120	93		
Level of understanding of clinical trials				51.215	<0.001
Good	182	133	49		
Moderate	140	61	79		
Poor	101	33	68		
History of Clinical Trials				27.641	<0.001
Yes	57	49	8		
No	366	178	188		
History of clinical trials among relatives and friends				12.387	<0.001
Yes	28	24	4		
No	395	203	192		
When participating in clinical trials, seek advice from relatives and friends				9.724	0.002
Yes	320	158	162		
No	103	69	34		
Understanding the functions of an ethics committee				45.980	<0.001
Yes	189	136	53		
No	234	91	143		

### 3.3 Results of the logistic regression analysis on predictors of willingness to participation among healthy subjects

The willingness to participate was a binary variable, with the assignment: “0” = non-willingness to participate, “1” = willingness to participate. Variables with statistically significant results in the univariate preliminary screening and those considered meaningful based on experience were included in the Logistic regression model, with a total of 11 variables included. Logistic regression identified three significant predictors: urban residence (*OR* = 2.175, *95%CI*: 1.291–3.664), being unemployed individuals not actively seeking work (*OR* = 2.891, *95%CI*: 1.134–7.371), and having a moderate understanding of clinical trials (*OR* = 3.906, *95%CI*: 1.950–7.827). Interestingly, awareness of ethics committee functions was associated with lower willingness (*OR* = 0.565, *95%CI*: 0.335–0.952). Other factors, such as age, gender, education, income, prior trial experience, and advice from relatives, were not statistically significant (see Table 3).

**Table 3:** Results of the logistic regression analysis on predictors of willingness to participation among healthy subjects.

Variable	Variable assignment	*B*	SE	*χ^2^*	*P*-value	OR (95%CI)
Constant		−0.336	0.994	0.114	0.735	0.715
Age	Continuous variable	0.036	0.020	3.202	0.074	1.036(0.997–1.077)
Gender	Female = 0, Male = 1	−0.379	0.258	2.158	0.142	0.685(0.413–1.135)
Residence	Rural = 0, Urban = 1	0.777	0.266	8.529	**0.003**	2.175(1.291–3.664)
Education level	Dummy variable					
Senior high school		0.212	0.561	0.143	0.705	1.236(0.412–3.710)
College and above		0.510	0.447	1.300	0.254	1.665(0.693–3.999)
Average monthly income (yuan)	Dummy variable					
3,000 ~ 4,999		0.628	0.338	3.467	0.063	1.875(0.967–3.633)
≥5,000		0.504	0.274	3.393	0.065	1.656(0.968–2.831)
Employment	Dummy variable					
Seeking employment		−0.131	0.311	0.179	0.673	0.877(0.477–1.613)
Unemployed individuals not actively seeking work		1.062	0.478	4.941	**0.026**	2.891(1.134–7.371)
Level of understanding of clinical trials	Dummy variable					
Moderate		1.363	0.355	14.769	**<0.001**	3.906(1.950–7.827)
Poor		0.393	0.319	1.526	0.217	1.482(0.794–2.767)
History of clinical trials	No = 0, Yes = 1	−0.750	0.469	2.563	0.109	0.472(0.189–1.183)
History of clinical trials among relatives and friends	No = 0, Yes = 1	−1.041	0.626	2.768	0.096	0.353(0.104–1.204)
When participating in clinical trials, seek advice from relatives and friends	No = 0, Yes = 1	0.407	0.273	2.213	0.137	1.502(0.879–2.567)
Understanding the functions of an ethics committee	No = 0, Yes = 1	−0.571	0.266	4.593	**0.032**	0.565(0.335–0.952)

Bold value indicates *p* < 0.05.

## 4 Discussion

Clinical drug trials represent a critical phase in pharmaceutical development, and healthy subjects serve as one of the essential participants in these studies. Understanding the willingness of potential healthy subjects to participate, clarifying the factors influencing their willingness, and implementing targeted measures to enhance their participation are crucial for improving clinical trial enrollment rates.

This survey found that the willingness of potential healthy subjects to participate in clinical trials was 53.66%, which is higher than the results reported by Chu et al. (9) in South Korea, significantly lower than the findings of Almutairi et al. (10) and Bouida et al. (11), and similar to the results of Raheja et al. (12) The considerable variation in participation rates across different regions and countries may be related to factors such as fear of investigational drugs or devices, lack of time, as well as social customs and cultural differences. Therefore, conducting region-specific investigations is essential to better enhance the participation rate of healthy subjects in clinical trials.

Unlike patient subjects who are motivated by potential clinical benefits, healthy subjects generally exhibit lower willingness to participate in trials since they do not seek therapeutic gains (13–15). Currently, most healthy subjects participating in clinical trials are individuals in need of financial compensation and with ample spare time, such as the unemployed individuals not actively seeking work or university students. Furthermore, the willingness of healthy subjects to participate is also influenced by the type of trial; for example, willingness is significantly lower for trials involving invasive procedures or those with serious side effects compared to non-invasive studies (16). Although this study did not differentiate among types of potential trial projects, practical experience has shown that participation rates tend to be higher for low-risk studies, such as dermal tests and other non-invasive, non-oral drug administration trials.

Our findings indicate that place of residence, employment status, and level of understanding of clinical trials serve as significant predictors of participation willingness. The results demonstrated that urban healthy subjects exhibited a significantly higher willingness to participate in clinical trials compared to those from rural areas. This discrepancy may be attributed to the more accessible dissemination of clinical trial knowledge and health education in urban settings, where individuals are more likely to be exposed to relevant information, leading to greater awareness and understanding of clinical trials (17). Complementarily, urban areas are typically endowed with more abundant medical resources and a higher concentration of clinical trial institutions, which enhances participation convenience while reducing associated time and transportation costs. Beyond structural advantages, urban populations often exhibit more open attitudes toward novel experiences and innovations, translating to a greater propensity to engage in clinical trials.

Furthermore, this urban–rural disparity is underpinned by structural socioeconomic determinants: urban populations generally demonstrate higher aggregate health literacy and educational attainment, which facilitate the comprehension of trial protocols and nuanced risk benefit assessments. Concurrently, differential access to digital information and technology – often substantially limited in rural settings – may constrain awareness of trial opportunities and reinforce informational inequities. Finally, variations in institutional trust and social capital between urban and rural communities could modulate perceptions of safety and willingness to engage in formal clinical research endeavors.

Our study revealed that unemployed individuals not actively seeking work healthy subjects demonstrated a higher willingness to participate compared to their employed counterparts, which contrasts with the findings reported by Rein-Hedin et al. (2) This discrepancy may be explained by the fact that most unemployed individuals not actively seeking work individuals lack a stable income and have more flexible schedules, enabling them to accommodate the time requirements of clinical trials more easily and reducing conflicts arising from employment obligations. Furthermore, some unemployed individuals not actively seeking work individuals may experience financial pressures, making the financial compensation offered for trial participation a motivating factor (15). Consequently, they may be more inclined to accept potential risks associated with clinical trials, thereby increasing their willingness to participate. This study found no evidence that healthy subjects actively seeking employment exhibited a higher willingness to participate. While both unemployed individuals not actively seeking work and those actively seeking employment lack permanent jobs, healthy subjects in the job-seeking group typically prioritize job-seeking as their primary focus and allocate their time and energy accordingly. They may be concerned that participating in clinical trials could interfere with their job search process, which could contribute to their lower willingness to participate.

The present study found that healthy subjects with a moderate level of awareness of clinical trials showed a significantly higher willingness to participate compared to those with lower levels of understanding. This is inconsistent with the research results of Elshammaa et al. (18), which may be related to cultural customs. When individuals have a clearer understanding of the objectives, procedures, potential risks, and benefits of clinical trials, they are better equipped to conduct a rational assessment of the advantages and disadvantages of involvement, thereby increasing their willingness to enroll. In contrast, those with limited awareness are more likely to hold misconceptions or concerns, such as fears about potential physical harm from investigational drugs or doubts regarding the safety of trial procedures, which may diminish their inclination to participate. It is noteworthy that while descriptive data suggested an association between high understanding and willingness, it did not emerge as a significant independent predictor in the multivariate model. This may indicate a non-linear relationship, where moderate knowledge optimally reduces uncertainty and fosters willingness, whereas high understanding may involve more complex, conditional decision-making that does not uniformly translate into higher participation intent.

Our study revealed a counterintuitive finding: healthy subjects aware of the functions of an ethics committee exhibited a lower willingness to participate, which contrasts with some prior studies conducted primarily in patient populations (19). This discrepancy may be explained by the unique perspective of healthy volunteers, who undertake risk without therapeutic benefit. For them, knowledge of ethical oversight might not merely signify protection, but also heighten the salience of potential risks and procedural burdens, leading to more cautious assessment. Furthermore, awareness could correlate with greater scrutiny of the informed consent process and the real-world independence of ethics committees, potentially uncovering reservations about their effectiveness in practice (20). This finding may also reflect a specific socio-cultural context where institutional trust is nuanced. Therefore, while ethical oversight is fundamental, simply increasing awareness of its existence may not be sufficient to bolster willingness among healthy subjects; instead, demonstrating its tangible effectiveness and commitment to participant welfare through transparent communication might be more critical.

Existing research suggests that younger (21), those with a history of clinical trial participation (22, 23), female (24), subjects with lower income levels (3), and those receiving recommendations from healthcare providers (8) are generally more inclined to participate in clinical trials. However, our study did not identify significant differences between the groups regarding these factors. This discrepancy may be attributable to the relatively small sample size and the narrow age distribution of the participants. Future multi-center studies with larger sample sizes are needed to better elucidate the influence of these factors on the willingness of healthy subjects to participate in clinical trials.

Although this study comprehensively discussed the factors affecting healthy subjects’ willingness to participate in clinical trials, it has several limitations. First, the cross-sectional design precludes causal inferences regarding the factors associated with willingness to participate. Second, the use of convenience sampling from a single geographic region limits the generalizability of the findings. Third, self-reported data are subject to social desirability and recall biases. Fourth, the assessment of willingness did not differentiate between types of clinical trials (e.g., invasive vs. non-invasive), which may influence real-world decisions. Finally, the sample size and concentrated age range may have limited the detection of some associations.

Despite these limitations, the study provides initial insights into an under-researched population in the Chinese context. Future studies with longitudinal designs, probability sampling, and differentiation of trial types are warranted to enhance causal inference and generalizability.

## 5 Conclusion

This study systematically elucidated the epidemiological characteristics of healthy subjects and the factors influencing their willingness to participate in clinical trials. The results indicated that urban residents, unemployed individuals not actively seeking work, and those with a moderate level of understanding of clinical trials exhibited a higher willingness to participate. In contrast, subjects unaware of the functions of an ethics committee showed lower willingness. These findings refine the characteristic profile of potential healthy subjects and provide a direct basis for developing targeted recruitment strategies, such as strengthening trial-related education in medically underserved communities and adopting differentiated communication to address specific information and motivation gaps.

Conducting comparative studies across different socioeconomic and cultural contexts, delving into the psychological mechanisms behind healthy subjects’ decisions to participate in clinical trials, and systematically designing and evaluating targeted recruitment programs represent critical steps for translating these insights into practice. Implementing these strategies may increase willingness among healthy subjects to engage in clinical trials, alleviate recruitment challenges, ensure the smooth progress of clinical trials, and ultimately promote the healthy development of pharmaceutical research.

## Data Availability

The raw data supporting the conclusions of this article will be made available by the authors, without undue reservation.
